# Effects of Heat Treatment Duration on the Electrical Properties, Texture and Color of Polymerized Whey Protein

**DOI:** 10.3390/molecules27196395

**Published:** 2022-09-27

**Authors:** Paulina Bielska, Dorota Cais-Sokolińska, Krzysztof Dwiecki

**Affiliations:** 1 Department of Dairy and Process Engineering, Faculty of Food Science and Nutrition, Poznań University of Life Sciences, ul. Wojska Polskiego 31, 60-624 Poznan, Poland; 2 Department of Food Biochemistry and Analysis, Poznań University of Life Sciences, ul. Mazowiecka 48, 60-623 Poznan, Poland

**Keywords:** electrical properties, zeta potential, conductivity, texture, color, whey protein, heat treatment

## Abstract

In this research effects of heat treatment duration on the electrical properties (zeta potential and conductivity), texture and color of polymerized whey protein (PWP) were analyzed. Whey protein solutions were heated for 30 min to obtain single-heated polymerized whey protein (SPWP). After cooling to room temperature, the process was repeated to obtain double-heated polymerized whey protein (DPWP). The largest agglomeration was demonstrated after 10 min of single-heating (zeta potential recorded as −13.3 mV). Single-heating decreased conductivity by 68% and the next heating cycle by 54%. As the heating time increased, there was a significant increase in the firmness of the heated solutions. Zeta potential of the polymerized whey protein correlated with firmness, consistency, and index of viscosity, the latter of which was higher when the zeta potential (r = 0.544) and particle size (r = 0.567) increased. However, there was no correlation between zeta potential and color. This research has implications for future use of PWP in the dairy industry to improve the syneretic, textural, and sensory properties of dairy products.

## 1. Introduction

Whey proteins (WPs), in addition to their nutritional value, are used in the food industry for their functional properties [1]. In native form the proteins have limited functionality, though this can be overcome by heat treatment to improve foaming, crosslinking, and emulsifying properties, among others [2]. Denaturation and aggregation of WPs has been thoroughly described in the literature [3,4,5]. Heat treatment being shown to lead to partial or complete unfolding of the tertiary conformation. This causes hydrophobic amine groups to be exposed, which increases protein volume and elasticity [6], with WPs heated above 70 °C forming gels or aggregates. In this regard, β-lactoglobulin content of WPs has a decisive influence on their ability to gel as it forms soluble polymers of high molecular weight [7].

WP aggregation is used in the food industry to improve its functional properties [1], and is dependent on the pH of the reaction medium. This is due to the reactivity and availability of the thiol group, with WPs being more resistant to denaturation in acidic environments than in alkaline solutions. Aggregation is also affected by the concentration of the protein in the heated solution as the process requires the collision of adjacent particles, so the higher the concentration, the faster the reaction [2]. Due to their ability to form aggregates, WPs give products shape and texture, which contributes to their overall sensory acceptability [8]. Soluble WP aggregates are polymerized whey protein (PWP) [2]. Reports suggest that the addition of these PWP improves the physicochemical and sensory properties of fermented milk [9,10,11]. For example, the addition of PWP to the production of goat’s milk yoghurt increased viscosity by 80% and reduced syneresis by up to 25% [12]. In addition, the functional properties of PWP are similar to salt, as they were able to thicken cheese mass by binding the ingredients due to their ability to emulsify fat and having high water absorption [13].

Rheological properties, stability, and texture of food depend on particle-particle and particle-media interactions. One of the most useful parameters for understanding electrical charge interactions in food is zeta potential (ZP) [14]. ZP is considered a predictor of the macroscopic behavior of the emulsion [15] and is a measure of the intensity of repulsion or attraction between particles. It is believed that the higher the absolute value of ZP, the more stable the emulsion [16]. Therefore, ZP can be used to optimize the formulas of suspensions and emulsions, as well as being a useful tool for predicting their stability [17]. In dairy research, ZP is most often used as an indicator of the electrical charge of milk fat globules, casein micelles [18], WP [19], and protein-probiotic interactions [20].

Protein aggregation requires understanding of the interactions between electrical charges. Therefore, the use of WP aggregates in food design prompted the analysis of the effects of heat treatment duration on the electrical properties of PWP, including ZP and conductivity. The polymerization process also changes the color and texture of WPs, therefore a comprehensive assessment of color was undertaken based on the measurement of chroma (C*) and indexes: whiteness index (WI), yellowness index (YI) or browning index (BI). In addition, the texture parameters that determine the quality and further use of PWP were analyzed. Literature data show that thermal behavior of whey protein depends on pH, protein concentration, temperature and time of heat treatment [2]. PWP are obtained by heating WPs at the temperature of 85 °C for 30 min (maintaining a constant pH of 7.0), and then cooling to room temperature [6,9]. In our study we analyzed the electrical properties, texture and color of polymerized whey protein also if we repeat the process again, therefore the same conditions were used for both heat treatment cycles. Use of the conditions for both cycles is important in the further use of polymerized whey protein. This can make a difference when the experiment is moved from the laboratory to the dairy industry.

## 2. Results and Discussion

### 2.1. Electrical Properties and Particle Size

**Figure 1:**
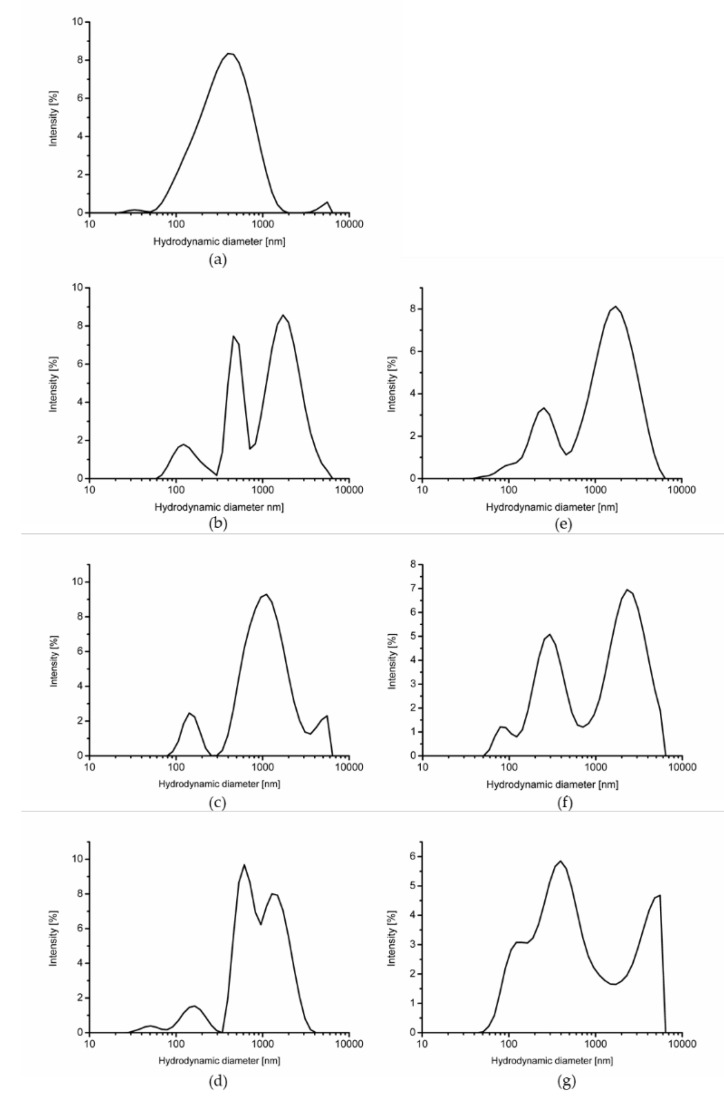
Particle size of polymerized whey protein depending on heating time (**a**): 0 min of heat treatment; (**b**–**d**): single-heated polymerized whey protein after 10, 20, and 30 min of heat treatment, respectively.; (**e**–**g**): double-heated polymerized whey protein after 10, 20, and 30 min of heat treatment, respectively.

ZP values made it possible to assess the surface charge and dispersion stability, with the protein particle’s agglomeration capacity initially recorded as −16.3 mV (Table 1). Differences in ZP values at the same pH value of the heated solutions most probably resulted from the different structure of the electrical layer formed around the agglomerates, as the ZP is a characteristic value of the surface in contact with the solution. Heating WP preparations caused significant changes in particle size distribution, with the largest agglomeration demonstrated after 10 min of single-heating (−13.3 mV). Approaching zero proves that protein polymers are covalently bonded to hydronium ions, and at that time there was a shift in particle size towards larger particles (from 301.5 to 627.8 nm; Table 1 and Figure 1). This demonstrates that the agglomeration process, and the largest aggregates formed after this time, had a size of 768.5 nm after 20 min of heating. Following double-heating, the agglomeration ability of the proteins decreased (*p* < 0.05), as the proteins with the lowest ability to agglomerate were those double-heated at 10 and 20 min (−18.4 mV). Furthermore, aggregates produced at the end of double-heating were 409.7 nm in size and were 1.5-times smaller than after 10 min of single-heating (627.8 nm, *p* < 0.05).

According to Zhao and Xiao [21], as pH increases, ZP decreases. The same authors also demonstrated that higher ZP values may result from denaturation of the WP. Sun et al. [22] analyzed the interactions between soy lecithin and WP, including WP after the polymerization process, and showed that the ZP of WP increased linearly with an increase in the level of soy lecithin, from 0 to 3%. According to the authors, the interactions may involve hydrophobic and electrostatic forces that caused changes in the functional and physicochemical properties of the WP. Jiang et al. [6] suggested that the ZP of WP after the polymerization process were lower than those of native WP under the same conditions, and that heating clearly induced the formation of large protein aggregates through a polymerization process that produces a stable and homogeneous network. Research by Khan et al. [1] suggests the use of 3,3′-diindolilometan to stabilize PWP as it resulted in a significant increase in mean particle size and highly negative ZP (±30 mV), indicating a stable solution due its electrostatic repulsive forces.

Heating WPs decreased conductivity by 68% (SPWP) and the next heating step by 54% (DPWP; *p* < 0.05) (Table 1). According to Guo and Xiong [23], there is a linear relationship between conductivity and final protein concentration. Electrical conductivity is a parameter influenced not only by composition and soluble salt fraction, but also by temperature [24]. According to Jambrak et al. [25], the decrease in conductivity is due to the presence of ion aggregates that are not involved in the conductivity process, and a lower conductivity is noted with increasing viscosity.

### 2.2. Color

**Figure 2:**
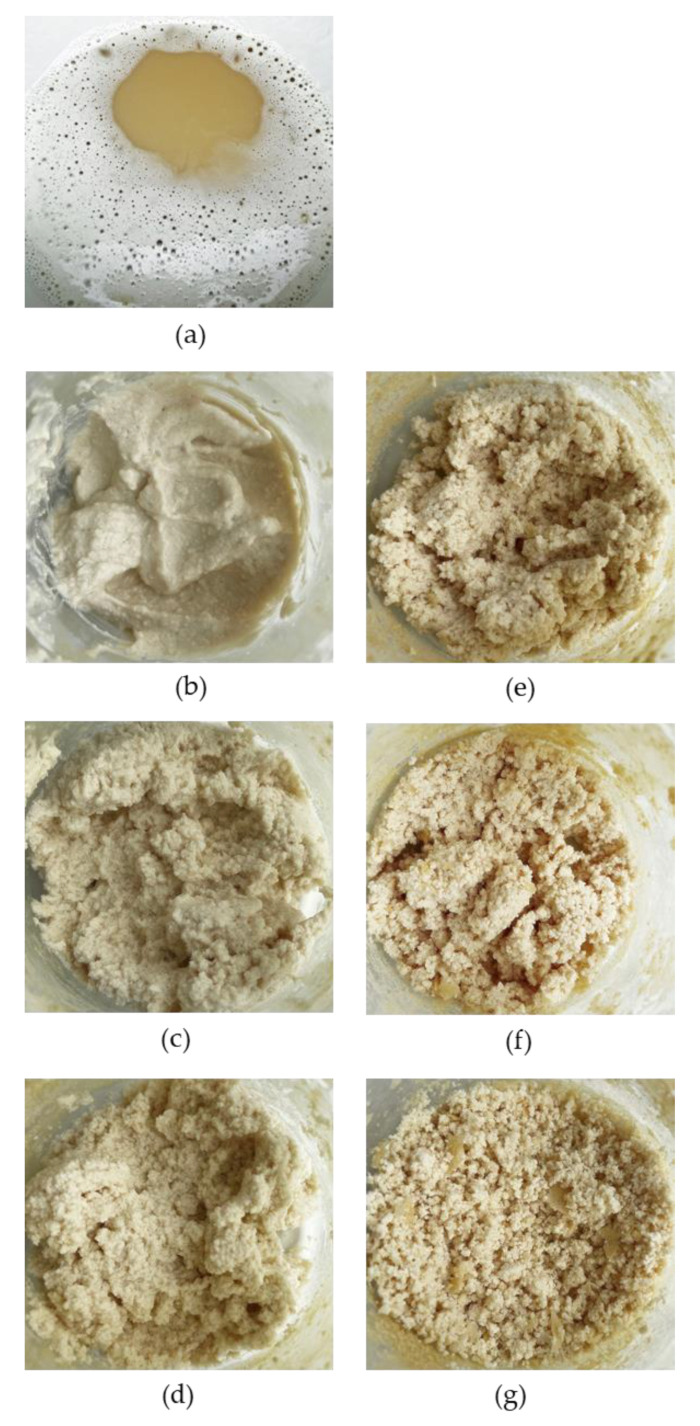
Polymerized whey protein depending on heating time. (**a**): 0 min time of heat treatment; (**b**–**d**): single-heated polymerized whey protein after 10, 20, or 30 min heat treatment, respectively; (**e**–**g**): double-heated polymerized whey protein after 10, 20, or 30 min heat treatment, respectively.

Protein aggregation is associated with turbidity and color change of heated solutions [26], hence the importance of color profiling based on the measurement of coordinates, chroma, and indexes: whiteness index, yellowness index or browning index. Decrease in lightness (parameter L*) in combination with the increase in parameter a* is a significant indicator of the intensity of browning of the heated colloid system associated with the Maillard reaction (MR). The heated solutions showed an increase of 46% in the lightness parameter with time of heat treatment (after 30 min) for SPWP (*p* < 0.05) (Table 2 and Figure 2). However, no differences in lightness were found in the time of heat treatment for DPWP (*p* > 0.05). Solutions became whiter as the heating time increased, but only in the case of SPWP. Single-heating caused a 1.3-fold increase in whiteness (*p* < 0.05), though there were no differences in the whiteness index during DPWP heating (*p* > 0.05). With increased heating time, an increase in the yellowness index, chroma and browning index parameters for the SPWP and DPWP was shown (*p* < 0.05). Color measurement is an important quality determinant, as is used to optimize and select the conditions of the technological process. Polymerized WP are introduced in the production of fermented milk to improve syneretic, textural and sensory properties [9,27]. However, Ortega et al. [28] suggest that the color of polymerized whey products correlates with antioxidant activity due to the MR.

### 2.3. Texture and Microstructure

Analysis of the firmness in the process of obtaining SPWP showed no statistically significant differences after 10 min of heating WP solutions (*p* > 0.05) (Table 3). However, as the heating time increased, there was a significant increase in the firmness of the heated solutions. After a further 20 min, firmness increased 10-fold (*p* < 0.05). A similar relationship was demonstrated by analyzing consistency, with no difference in consistency after 10 min of heating (*p* > 0.05), however an additional 20 min of heating resulted in a 5-fold increase in this parameter (*p* < 0.05). There was no difference in firmness or consistency in the subsequent stages of heating (obtaining DPWP) (*p* > 0.05). Cohesiveness increased as a result of single-heating (*p* < 0.05), though the next step of heating (30 min, preparation of DPWP) caused an 11-fold reduction in this parameter (*p* < 0.05). Jiang et al. [6] and Zhang et al. [2] showed that the viscosity of PWP increased with increase in temperature and protein concentration. The same relationships were found in the index of viscosity analysis, but only for the single-heating process. Indeed, the next stage of heating caused a significant reduction in this parameter (*p* < 0.05).

Analysis of PWP texture parameters is important for the incorporation of PWP into dairy products and their design quality. Bierzuńska et al. [9] suggest that the introduction of PWP to the processing milk used in the production of yoghurt causes a 3-fold increase in the viscosity index of the finished product. According to Wang et al. [11] PWP (0.3%, *w*/*v*) interacts with the casein network to create a dense structure, and in combination with low-methoxyl pectin (0.2%, *w*/*v*), improves the physical properties of kefir. PWP is also used as a fat substitute in food products [2,10] and as a thickener [27]. The heating time of PWP is also gaining importance, with Gustaw [29] demonstrating that yoghurts with the addition of 1% PWP obtained as a result of single or double-heating have similar rheological properties. However, yoghurt with the addition of PWP heated for longer was characterized by greater hardness. Author also suggests that the heating time in the polymerization process affects the content of whey protein polymers in the solution. Glibowski et al. [30] suggest that the use of single and double heating has an influence on the preparation of protein gels using calcium ions as inducing agents. According to the authors, double heating solutions gel faster, with lower concentrations of protein and calcium ions and texture of protein gels depends on the presence of calcium ions.

The previously suggested relationship between rheological properties and conductivity [25] was the rationale behind the analysis of the relationship between texture parameters, electrical properties, and particle size of PWP due to heating time. A high inverse correlation of conductivity (*p* < 0.05) from firmness and consistency was demonstrated (−0.760 and −0.847; Table 4). Furthermore, conductivity correlated with lightness and all of the calculated color indices, with correlation coefficients ranging from −0.824 to −0.944. ZP was not related to color (Table 4), though it was correlated with texture parameters including, firmness, consistency, and index of viscosity (r respectively −0.715; −0.579 and 0.544). It was therefore shown that the greater the ZP, the greater the index of viscosity. Also, a directly proportional relationship was demonstrated between the index of viscosity and Z-average (r = 0.567).

**Figure 3:**
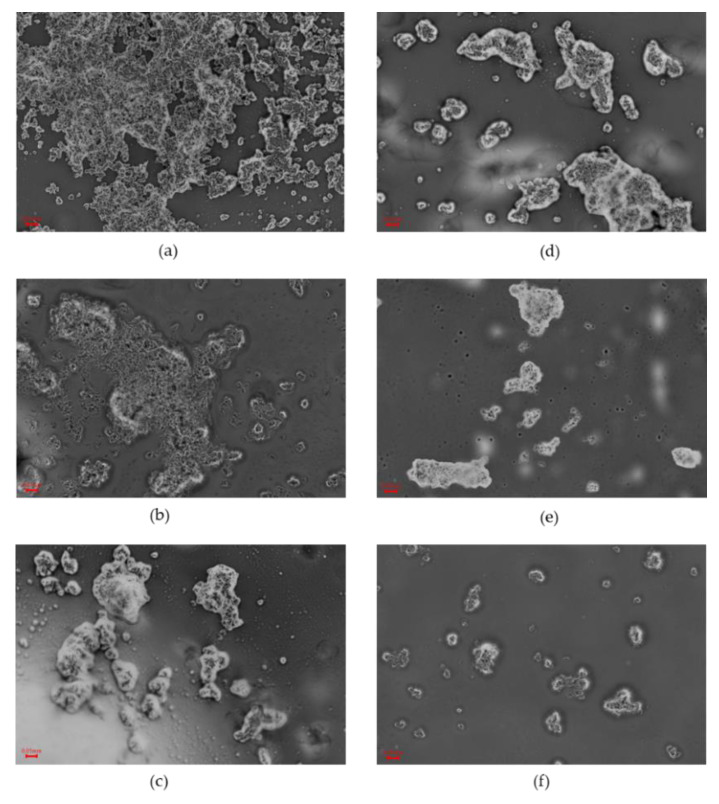
Microstructure of polymerized whey protein depending on the time of heat treatment. (**a**–**c**): single-heated polymerized whey protein after 10, 20, or 30 min, respectively; (**d**–**f**): double-heated polymerized whey protein after 10, 20, or 30 min, respectively; optical microscopy, 40× objective.

Microscopic images of the samples showed the formation of aggregates in the heated solutions. Along with a longer heating time in the process of obtaining SPWP, an increased agglomeration capacity of WP was observed. (Figure 3a–c). Subsequent heating steps during the preparation of DPWP caused the agglomeration ability of the protein to decline (Figure 3d–f). Thus, the microscopic images reflect the instrumental measurement of the texture of the PWP.

## 3. Materials and Methods

### 3.1. Polymerized Whey Protein

Preparation of PWP (28%, *w*/*v*) was carried out as follows: WPC80 whey protein concentrate powder obtained from whey after the production of rennet cheese (Mlekovita, Wysokie Mazowieckie, Poland) was dissolved in cold purified water and allowed to stand at 4 °C for 12 h. WPC dispersion was adjusted to pH 7.0 using 0.1 M sodium hydroxide at 21 °C. SPWP was heated at 85 °C for 30 min and then rapidly cooled to room temperature in ice-water under agitation [9]. Solutions were then heated again, keeping exactly the same conditions as used in the first heating process. Further heating at 85 °C for 30 min led to the formation of DPWP, which was then rapidly cooled to room temperature in ice-water under agitation. The use of cooling to room temperature separates the cycle of heating and makes the heating conditions of the second cycle the same to those of the first cycle (maintaining a constant pH of 7.0) according to the methodology [29]. The second cycle of heat treatment was started immediately after cooling to room temperature. Cooling time after each heat treatment cycle was no longer than 15 min. During heating, samples were taken for analysis every 10 min. 

### 3.2. Particle Size and Zeta Potential

Particle size (hydrodynamic diameter) was determined by dynamic light scattering and ZP was measured by electrophoretic light scattering using a Zetasizer Nano ZS-90 (Malvern Instruments Ltd., Malvern, UK). ZP values were presented as the arithmetic mean of 6 independent measurements. Particle size was presented as z-average (intensity weighted mean hydrodynamic diameter) and particle size distribution by intensity. The polydispersity index was also calculated. Particle size results were based on at least 3 independent measurements. A constant pH of 7.0 was used.

### 3.3. Electrical Conductivity

Electrical conductivity was measured using a CP–402 conductivity meter equipped with an EC-60 electrode (Elmetron, Zabrze, Poland). The volume of the sample was 25 cm^3^. During heating, samples were taken for analysis every 10 min.

### 3.4. Color

Instrumental color measurement was based on the CIELAB color space described by Cais-Sokolińska et al. [31]. Measurements were performed with a geometry SPIN using an X-Rite SP-60 camera (X-Rite, Grandville, MI, USA) equipped with a spherical geometry (diffusive), and a measurement chamber with a DRS-811 ceramic insert. The camera was calibrated based on the white and black reference standards SP-62-162 (X-Rite, Grandville, MI, USA). The whiteness index (WI), yellowness index (YI), chroma (C*) and browning index (BI) were calculated using the equation:

WI = 100 − [(100 − L*)^2^ + a*^2^ + b*^2^]^0.5^ (1)

YI = 142.86b* · L*^−1^ (2)

C* = (a*^2^ + b*^2^)^0.5^ (3)

BI = [100(x − 0.31)] · 5.88 where x = (a* + 1.750 · L*) · (5.645 · L* + a* − 3.012 · b*)^−1^ (4)

### 3.5. Texture

Firmness, consistency, cohesiveness and viscosity index of the fermented samples were determined by reverse extrusion in a TA-XTplus texture meter (Stable Micro Systems, Surrey, UK) [9], with use of an A/BE attachment and compression disc (Ø = 35 mm). Samples were placed inside a cylinder with an internal diameter of Ø = 50 mm (75% filling). Measurement conditions were a distance of 30 mm, pretest 1.0 mm/s and post-test 10.0 mm/s. Results were recorded in Texture Exponent E32 version 4.0.9.0 software (Godalming, Surrey, UK).

### 3.6. Microstructure

Observations of microstructure were conducted on samples deposited onto a glass slide, and covered with a cover slip, for observation under a ProteOne optical microscope (Delta Optical, Mińsk Mazowiecki, Poland). Observations were made at 40× magnification using a ProteOne semi-plan achromatic objective (Delta Optical, Mińsk Mazowiecki, Poland) with oil immersion. Images were taken using a DLTCam PRO microscope camera (Delta Optical, Mińsk Mazowiecki, Poland).

### 3.7. Statistical Evaluation

Verification of statistical hypotheses was achieved using a level of significance of α = 0.05. The influence of the composition and storage time on the samples was evaluated by two-way analysis of variance followed by Tukey’s HSD post hoc test for multiple comparisons. Data were analyzed using Statistica data analysis software, version 13 (TIBCO Software Inc., Palo Alto, CA, USA).

## 4. Conclusions

Determining the zeta potential value is related not only to the determination of the surface charge, but also the dispersion stability of the heated solutions. Based on the electrical properties of the polymerized whey protein, it is possible to estimate the texture properties and color indices depending on the heating time. Duration of heat treatment has an effect on a change in the particle size distribution, which translates into a change in its color and texture. This has implications for future use of polymerized whey protein in the dairy industry to improve the syneretic, textural, and sensory properties of dairy products.

## Figures and Tables

**Table 1:** Electrical properties and particle size of polymerized whey protein depending on the heating time.

Parameters	Sample	Time of Heated (min)				MSE
		0	10	20	30	
Zeta potential (mV)	SPWP	−16.3 ± 0.1 ^cB^	−13.3 ± 0.3 ^aA^	−17.0 ± 0.2 ^dA^	−15.8 ± 0.2 ^bA^	0.03750
	DPWP	−15.8 ± 0.2 ^aA^	−18.4 ± 0.1 ^cB^	−18.4 ± 0.2 ^cB^	−17.4 ± 0.3 ^bB^	0.03167
Conductivity (mS/cm^−1^)	SPWP	2.989 ± 0.0 ^dB^	2.004 ± 0.0 ^cB^	1.421 ± 0.0 ^bB^	0.967 ± 0.2 ^aB^	0.01034
	DPWP	0.967 ± 0.2 ^bA^	0.790 ± 0.0 ^bA^	0.449 ± 0.0 ^aA^	0.448 ± 0.0 ^aA^	0.01031
Z-average (d, nm)	SPWP	301.5 ± 5.1 ^aA^	627.8 ± 142.7 ^bA^	768.5 ± 93.4 ^bB^	656.0 ± 61.3 ^bB^	8219.3
	DPWP	656.0 ± 61.3 ^bB^	673.1 ± 79.4 ^bA^	525.2 ± 106.3 ^abA^	409.7 ± 67.4 ^aA^	6474.3

MSE: mean square error of inter-group variability; SPWP: single-heated polymerized whey protein; DPWP: double-heated polymerized whey protein; a–d, A,B: different small letters in superscript in the rows and capital letters between SPWP and DPWP indicate statistically significant differences at the level *p* = 0.05.

**Table 2:** Assessment of color polymerized whey protein depending on the heating time.

Assessment of Color	Sample	Time of Heated (min)				MSE
		0	10	20	30	
L*	SPWP	56.99 ± 0.2 ^aA^	78.25 ± 0.1 ^bA^	80.62 ± 0.1 ^cA^	83.09 ± 0.0 ^dA^	0.01127
	DPWP	83.09 ± 0.0 ^aB^	85.47 ± 0.2 ^aB^	83.84 ± 0.4 ^aB^	83.59 ± 2.0 ^aA^	1.06600
WI	SPWP	55.81 ± 0.2 ^aA^	72.35 ± 0.0 ^bA^	74.82 ± 0.0 ^cB^	74.87 ± 0.0 ^cA^	0.01519
	DPWP	74.87 ± 0.0 ^aB^	74.60 ± 0.1 ^aB^	74.01 ± 0.1 ^aA^	73.41 ± 1.2 ^aA^	0.39021
YI	SPWP	25.32 ± 0.6 ^aA^	30.13 ± 0.0 ^cA^	27.36 ± 0.3 ^bA^	30.63 ± 0.1 ^cA^	0.08141
	DPWP	30.63 ± 0.1 ^aB^	33.27 ± 0.0 ^bB^	33.14 ± 0.1 ^bB^	34.17 ± 0.8 ^bB^	0.18144
C*	SPWP	10.18 ± 0.2 ^aA^	17.08 ± 0.0 ^cA^	16.08 ± 0.0 ^bA^	18.59 ± 0.0 ^dA^	0.00962
	DPWP	18.59 ± 0.0 ^aB^	20.84 ± 0.0 ^cB^	20.35 ± 0.1 ^bB^	20.88 ± 0.0 ^cB^	0.00485
BI	SPWP	20.70 ± 0.5 ^aA^	27.38 ± 0.0 ^cA^	24.94 ± 0.0 ^bA^	28.39 ± 0.1 ^dA^	0.06402
	DPWP	28.39 ± 0.1 ^aB^	31.34 ± 0.0 ^bB^	31.17 ± 0.1 ^bB^	32.19 ± 0.9 ^bB^	0.20496

L*: lightness; WI: whiteness index; YI: yellowness index; C*: chroma; BI: browning index; a–d, A,B: different small letters in superscript in the rows and capital letters between SPWP and DPWP indicate statistically significant differences at the level *p* = 0.05.

**Table 3:** Texture parameters polymerized whey protein depending on the heating time.

Texture Parameters	Sample	Time of Heated (min)				MSE
		0	10	20	30	
Firmness (g)	SPWP	14.21 ± 0.5 ^aA^	87.29 ± 5.7 ^aA^	505.25 ± 90.3 ^bA^	842.18 ± 101.6 ^cA^	4623.8
	DPWP	842.18 ± 101.6 ^aB^	1991.79 ± 751.4 ^aB^	1418.32 ± 739.7 ^aB^	1213.29 ± 494.7 ^aB^	3417 × 10^2^
Consistency (g/s)	SPWP	267.66 ± 16.3 ^aA^	2012.23 ± 296.3 ^aA^	6988.43 ± 1411.6 ^bA^	10,724.64 ± 1081.4 ^cA^	8125 × 10^2^
	DPWP	10,724.64 ± 1081.4 ^aB^	13,915.35 ± 3804.9 ^aB^	10,324.64 ± 3732.2 ^aB^	9577.46 ± 2836.3 ^aA^	9405 × 10^3^
Cohesiveness (g)	SPWP	8.68 ± 0.8 ^aA^	89.18 ± 10.8 ^aA^	265.71 ± 81.0 ^bB^	348.50 ± 19.1 ^bB^	1761.0
	DPWP	348.50 ± 19.1 ^bB^	126.00 ± 87.2 ^aA^	64.71 ± 44.7 ^aA^	30.82 ± 8.5 ^aA^	2507.6
Index of viscosity (g/s)	SPWP	22.53 ± 0.7 ^aA^	184.67 ± 13.8 ^abB^	246.00 ± 43.4 ^bB^	311.87 ± 83.6 ^bB^	2266.5
	DPWP	311.87 ± 83.6 ^bB^	20.11 ± 14.3 ^aA^	12.22 ± 10.9 ^aA^	4.23 ± 1.7 ^aA^	1830.1

a–c, A,B: different small letters in superscript in the rows and capital letters between SPWP and DPWP indicate statistically significant differences at the level *p* = 0.05.

**Table 4:** Correlation analysis of electrical properties and particle size of polymerized whey protein depending on the heating time on its texture and color.

Parameters		Conductivity (mS/cm^−1^)	Zeta Potential (mV)	Z-Average (d, nm)
Firmness (g)	r	−0.760	−0.715	0.169
	*p*	0	0	0.431
Consistency (g/s)	r	−0.847	−0.579	0.375
	*p*	0	0	0.071
Cohesiveness (g)	r	−0.236	0.199	0.639
	*p*	0.266	0.352	0.001
Index of viscosity (g/s)	r	0.035	0.544	0.567
	*p*	0.870	0.006	0.004
L*	r	−0.902	−0.249	0.574
	*p*	0	0.241	0.003
WI	r	0.824	0.125	−0.671
	*p*	0	0.561	0
YI	r	−0.869	−0.387	0.103
	*p*	0	0.062	0.632
C*	r	−0.944	−0.363	0.349
	*p*	0	0.081	0.094
BI	r	−0.912	−0.386	0.205
	*p*	0	0.063	0.338

r: correlation coefficient; *p*: the significance of the correlation coefficient.

## Data Availability

All data generated or analyzed during this study are available upon request from the author.

## Sample Availability

Samples of the compounds are available from the authors upon reasonable request.

## References

[1] Khan A., Wang C., Sun X., Killpartrick A., Guo M. (2019). Physicochemical and microstructural properties of polymerized whey protein encapsulated 3,3′-diindolylmethane nanoparticles. Molecules.

[2] Zhang X., Sun X., Gao F., Wang J., Wang C. (2019). Systematical characterization of physiochemical and rheological properties of thermal-induced polymerized whey protein. J. Sci. Food Agric..

[3] Chevallier M., Riaublanc A., Lopez C., Hamon P., Rousseau F., Croguennec T. (2016). Aggregated whey proteins and trace of caseins synergistically improve the heat stability of whey protein-rich emulsions. Food Hydrocoll..

[4] Joyce A.M., Kelly A.L., O’Mahony J.A. (2018). Controlling denaturation and aggregation of whey proteins during thermal processing by modifying temperature and calcium concentration. Int. J. Dairy Technol..

[5] Li R., Lund P., Nielsen S.B., Lund M.N. (2022). Formation of whey protein aggregates by partial hydrolysis and reduced thermal treatment. Food Hydrocoll..

[6] Jiang S., Hussain M.A., Cheng J., Jiang Z., Geng H., Sun Y., Sun C., Hou J. (2018). Effect of heat treatment on physicochemical and emulsifying properties of polymerized whey protein concentrate and polymerized whey protein isolate. LWT.

[7] Vardhanabhuti B., Foegeding E.A., McGuffey M.K., Daubert C.R., Swaisgood H.E. (2001). Gelation properties of dispersions containing polymerized and native whey protein isolate. Food Hydrocoll..

[8] Mahomud M.S., Katsuno N., Nishizu T. (2017). Formation of soluble protein complexes and yoghurt properties influenced by the addition of whey protein concentrate. Innov. Food Sci. Emerg. Technol..

[9] Bierzuńska P., Cais-Sokolińska D., Yiğit A. (2019). Storage stability of texture and sensory properties of yogurt with the addition of polymerized whey proteins. Foods.

[10] Fang T., Shen X., Hou J., Guo M. (2019). Effects of polymerized whey protein prepared directly from cheese whey as fat replacer on physiochemical, texture, microstructure and sensory properties of low-fat set yogurt. LWT.

[11] Wang H., Wang C., Guo M. (2021). Impact of polymerized whey protein/pectin thickening (PP) system on physical properties and volatile compounds of goat milk kefir mild and kefir. J. Food Sci..

[12] Li J., Guo M. (2006). Effects of polymerized whey proteins on consistency and water-holding properties of goat’s milk yogurt. J. Food Sci..

[13] Sołowiej B.G., Nastaj M., Szafrańska J.O., Muszyński S., Gustaw W., Tomczyńska-Mleko M., Mleko S. (2020). Effect of emulsifying salts replacement with polymerised whey protein isolate on textural, rheological and melting properties of acid casein model processed cheeses. Int. Dairy J..

[14] Cano-Sarmiento C., Téllez-Medina D.I., Viveros-Contreras R., Cornejo-Mazón M., Figueroa-Hernández C.Y., García-Armenta E., Alamilla-Beltrán L., García H.S., Gutiérrez-López G.F. (2018). Zeta potential of food matrices. Food Eng. Rev..

[15] Ravindran S., Williams M.A.K., Ward R.L., Gillies G. (2018). Understanding how the properties of whey protein stabilized emulsions depend on pH, ionic strength and calcium concentration, by mapping environmental conditions to zeta potential. Food Hydrocoll..

[16] Zhang X., Qi B., Xie F., Hu M., Sun Y., Han L., Li L., Zhang S., Li Y. (2021). Emulsion stability and dilatational rheological properties of soy/whey protein isolate complexes at the oil-water interface: Influence of pH. Food Hydrocoll..

[17] Kaszuba M., Corbett J., Watson F.M.N., Jones A. (2010). High-concentration zeta potential measurements using light-scattering techniques. Philos. Trans. R. Soc. A Math. Phys. Eng. Sci..

[18] Tholstrup Sejersen M., Salomonsen T., Ipsen R., Clark R., Rolin C., Balling Engelsen S. (2007). Zeta potential of pectin-stabilised casein aggregates in acidified milk drinks. Int. Dairy J..

[19] Carter B.G., Drake M.A. (2021). Influence of oral movement, particle size, and zeta potential on astringency of whey protein. J. Sens. Stud..

[20] Doherty S.B., Gee V.L., Ross R.P., Stanton C., Fitzgerald G.F., Brodkorb A. (2011). Development and characterisation of whey protein micro-beads as potential matrices for probiotic protection. Food Hydrocoll..

[21] Zhao Z., Xiao Q. (2017). Effect of chitosan on the heat stability of whey protein solution as a function of pH. J. Sci. Food Agric..

[22] Sun X.M., Wang C.N., Guo M.R. (2018). Interactions between whey protein or polymerized whey protein and soybean lecithin in model system. J. Dairy Sci..

[23] Guo A., Xiong Y.L. (2021). Electrical conductivity: A simple and sensitive method to determine emulsifying capacity of proteins. J. Food Sci..

[24] Ayadi M.A., Leuliet J.C., Chopard F., Berthou M., Lebouché M. (2004). Electrical conductivity of whey protein deposit: Xanthan gum effect on temperature dependency. Food Bioprod. Process..

[25] Jambrak A.R., Mason T.J., Lelas V., Herceg Z., Herceg I.L. (2008). Effect of ultrasound treatment on solubility and foaming properties of whey protein suspensions. J. Food Eng..

[26] Guo M., Wang H., Wang C. (2018). Interactions between whey protein and inulin in a model system. J. Food Sci. Technol..

[27] Arbab Sakandar H., Imran M., Huma N., Ahmad S., Khuram H., Aslam W., Azam M., Shoaib M. (2014). Effects of polymerized whey proteins isolates on the quality of stirred yoghurt made from camel milk. J. Food Process Technol..

[28] Ortega L., Romero A., Muro C., Riera F. (2015). Antioxidant activity and functional properties of polymerized whey products by glycation process. Int. J. Polym. Sci..

[29] Gustaw W. (2007). Effect of addition of whey protein aggregates obtained by single and double heating method on the rheological properties of set yoghurts. Pol. J. Food Nutr. Sci..

[30] Glibowski P., Mleko S., Wesolowska-Trojanowska M. (2006). Gelation of single heated vs. double heated whey protein isolate. Int. Dairy J..

[31] Cais-Sokolińska D., Pikul J., Wójtowski J., Danków R., Teichert J., Czyżak-Runowska G., Bagnicka E. (2015). Evaluation of quality of kefir from milk obtained from goats supplemented with a diet rich in bioactive compounds. J. Sci. Food Agric..

